# Tumor Cell‐Expressed Herpesvirus Entry Mediator Regulates Proliferation and Adaptive Immunity in Ovarian Cancer

**DOI:** 10.1002/iid3.70175

**Published:** 2025-03-19

**Authors:** Yun Lu, Yijun Zhang, Wenxuan Li, Haonan Jiang, Jiapo Wang, Xiaoqing Guo

**Affiliations:** ^1^ Shanghai Key Laboratory of Maternal Fetal Medicine, Shanghai Institute of Maternal‐Fetal Medicine and Gynecologic Oncology, Shanghai First Maternity and Infant Hospital, School of Medicine Tongji University Shanghai China; ^2^ Department of Gynecological Oncology, Shanghai First Maternity and Infant Hospital, School of Medicine Tongji University Shanghai China

**Keywords:** chemokines, herpesvirus entry mediator, innate and adaptive immunity, ovarian cancer, proliferation, STAT signal

## Abstract

**Background:**

Ovarian cancer (OvCa) is a prevalent gynecological malignancy with an increasing incidence and high mortality rate. Although the role of the herpesvirus entry mediator (HVEM), encoded by the *TNFRSF14* gene, is currently considered pivotal in various types of cancer, the regulation of tumor cell‐expressed HVEM in OvCa remains inadequately understood.

**Methods:**

Specimens were used to detect HVEM expression via quantitative RT‐PCR and flow cytometry. The proliferation of the murine OvCa cell line ID8 was determined using the Cell Counting Kit‐8, colony formation, and EdU staining assays. The immune constituents within the ascites fluid and spleen of tumor‐bearing mice were analyzed by flow cytometry. Bioinformatics analysis was performed to explore cytokines, chemokines, and signaling pathways regulated by HVEM, and differential expression levels were confirmed via quantitative RT‐PCR and western blot analysis.

**Results:**

Herein, we identified a significant upregulation of HVEM in OvCa tissues compared with that in benign tissues and observed dominant expression of HVEM in CD45⁻EpCAM⁺ subsets in OvCa specimens. Tumor cell‐expressed HVEM was found to promote OvCa cell proliferation by partly activating spliced X‐box‐binding protein 1 (XBP1s)‐c‐Myc signaling. In mouse models, knockdown of *Tnfrsf14* in ID8 cells alleviated OvCa progression and specifically affected the frequency and function of T cells in the ascites fluid and spleen. In addition, tumor cell‐expressed HVEM altered chemokine expression (CXCL1/9/10/11 and CCL2/4/5) and STAT signal activation (STAT5 and STAT6) in ID8 cells.

**Conclusion:**

This study investigated the effects of HVEM on OvCa and validated its potential as a therapeutic marker for treating OvCa.

## Graphical abstract

(1) Tumor cell‐expressed herpesvirus entry mediator (HVEM) was found to promote ovarian cancer (OvCa) cell proliferation by partly activating XBP1s‐cMyc signaling. (2) Tumor cell‐expressed HVEM specifically affected the effects of adaptive immunity and contributed to OvCa progression in mice. (3) Tumor cell‐expressed HVEM altered chemokine expression and the STAT signal activation in ID8 cells.

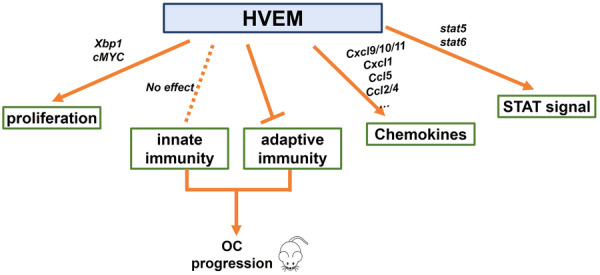

**Abbreviations**
AKT/PKB: protein kinase B
B7‐H3: B7 homolog 3
BRAC: breast cancer susceptibility gene
BRAF: v‐raf murine sarcoma viral oncogene homolog B
BTLA: B‐ and T‐lymphocyte attenuator
Ccl: C‐C motif chemokine
CD160: cluster of differentiation 160
CD45: cluster of differentiation 45
cMyc: Myc proto‐oncogene protein
Csf1r: colony stimulating factor 1 receptor
CTL: cytotoxic T lymphocyte
CTLA‐4: cytotoxic T‐lymphocyte protein 4
Cxcl: C‐X‐C motif chemokine
CXCR3: C‐X‐C motif chemokine receptor 3
DC: dendritic cell
EpCAM: epithelial cell adhesion molecule
ER: endoplasmic reticulum
Foxp3: forkhead box protein P3
Hck: HCK proto‐oncogene
HIF‐1α: hypoxia‐inducible factor 1‐alpha
HVEM: herpesvirus entry mediator
IFN‐γ: interferon‐gamma
Il15ra: interleukin 15 receptor subunit alpha
Il2rg: interleukin 2 receptor subunit gamma
IRE1: inositol‐requiring enzyme 1
JAK: janus kinase
LIGHT/TNFSF14: tumor necrosis factor superfamily member 14
Ltb: lymphotoxin beta
MDSC: myeloid‐derived suppressor cell
mTOR: mammalian target of rapamycin
NF‐κB: nuclear factor‐κB
NK: natural killer cell
NKT: natural killer T cell
OvCa: ovarian cancer
PD‐1: programmed death 1
PD‐L1: programmed cell death 1 ligand 1
Rac2: ras‐related C3 botulinum toxin substrate 2
STAT: signal transducer and activator of transcription
**T**_**EM**_
: effector memory T cell
TIM‐3: T cell immunoglobulin and mucin domain‐containing protein 3
TNFRSF14: tumor necrosis factor receptor superfamily member 14
TNF‐α: tumor necrosis factor‐alpha
Treg: regulatory T cell
VISTA: V‐type immunoglobulin domain‐containing suppressor of T‐cell activation
Was: WASP actin nucleation promoting factor
XBP1: X‐box binding protein 1

## 1 Introduction

Ovarian cancer (OvCa) is a prevalent gynecological malignancy with the third‐highest incidence and second‐highest mortality rate, according to global cancer statistics for 2022, which seriously endangers the physical and mental health of women [1]. OvCa can be subdivided into different subtypes, with epithelial cancer constituting approximately 90% of all cases [2]. Owing to the lack of an effective screening strategy, more than 70% of clinical patients present in advanced stages (Stage III or Stage IV) at the time of diagnosis, with widespread dissemination throughout the abdominal cavity [3]. The conventional approach for managing primary advanced OvCa is cytoreductive surgery, followed by chemotherapy with a platinum/taxane combination. However, relapse and the eventual development of platinum resistance pose considerable challenges to tumor management [4]. According to the current recommendations provided by the National Comprehensive Cancer Network, secondary cytoreduction is considered a viable treatment for recurrent OvCa, but only for a limited number of individuals [5]. In recent years, various tumor mutations, including the BRAF and BRAC1/2 mutation, have been identified, and targeted molecular inhibitors for treating OvCa have emerged, which are currently undergoing different clinical trials to evaluate their antitumor efficacy. In particular, poly (ADP‐ribose) polymerase inhibitors (PARPis) have emerged as critical maintenance therapies for OvCa patients harboring BRCA1/2 mutations which result in homologous recombination deficiency [6, 7]. Homologous recombination deficiency is usually connected with platinum‐based therapy and PARP inhibitors’ response, and it affects treatment decisions in both first‐line and second‐line therapy. Expanding the utility of biomarkers, and broadening the actionable biomarker repertoire are essential for advancing personalized treatment strategies [6]. Hence, it is important to understand the pathogenesis of OvCa and identify more effective diagnostic methods alongside new therapies for disease control.

The potential for biomarker development arises from the characteristics of identified tumor antigens, novel molecular tumor mutations [7], infiltration of immune cells into tumors, and the presence of immune checkpoint receptors and ligands, which represent significant breakthroughs in cancer immunotherapy [8, 9]. In 2011, the Food and Drug Administration approved ipilimumab, a monoclonal antibody that specifically targets cytotoxic T‐lymphocyte protein 4 (CTLA‐4), as the first immune checkpoint inhibitor [10]. Nivolumab, an immune checkpoint inhibitor blocking the signaling mediated by programmed death 1 (PD‐1) and its ligand PD‐L1, subsequently received Food and Drug Administration approval [11]. Several types of OvCa immunotherapies, including PD‐1/CTLA‐4 inhibitors, combined with cancer vaccines and/or chemotherapy, have been tested in clinical trials [12]. In addition, other potential targets of immune checkpoints (such as B7 homolog 3 [B7‐H3], B‐ and T‐lymphocyte attenuator [BTLA], and V‐type immunoglobulin domain‐containing suppressor of T‐cell activation [VISTA]) for anticancer therapies have emerged in recent studies [13, 14, 15].

The herpesvirus entry mediator (HVEM), also known as tumor necrosis factor receptor superfamily member 14 (TNFRSF14), belongs to the tumor necrosis factor receptor superfamily and comprises a signal peptide and three domains: extracellular, cytoplasmic, and transmembrane. The extracellular domain of HVEM, which interacts with its binding partners, contains four cysteine‐rich domains [16, 17]. Unlike other members of the TNFR superfamily, HVEM exhibits a distinct binding profile because it acts as both a receptor and ligand [18]. As a receptor, HVEM binds to the TNF superfamily cytokines lymphotoxin‐alpha and LIGHT; as a ligand, HVEM binds to the immunoglobulin superfamily receptors BTLA and cluster of differentiation 160 (CD160) [18, 19]. Moreover, HVEM can also form a complex with glycoprotein D [20]. HVEM is widely expressed on the surface of T, B, dendritic, and epithelial cells and contributes to the pathogenesis of several experimental mouse models and human diseases [18, 19, 21]. Dysregulated expression of HVEM has also been extensively reported in several types of cancer; however, survival rates and the efficacy of anticancer therapies remain inconclusive [17]. To date, our understanding of the role of HVEM in OvCa is limited. Elevated HVEM expression in ovarian serous adenocarcinoma tissues is correlated with clinicopathological features, such as TNM staging, lymph node metastasis, and recurrence [22]. Silencing HVEM in the human OvCa cell line OVCAR3 reportedly did not affect proliferation, early apoptosis, or cell cycle distribution [23] but inhibited cell proliferation and promoted cell apoptosis under hypoxic conditions [24]. Moreover, silencing HVEM in OVCAR3 was shown to increase T cell migration and enhance TNF‐α and interferon‐gamma (IFN‐γ) secretion in vitro under normoxic conditions, while promoting hypoxia‐inducible factor 1‐alpha (HIF‐1α) activity via the AKT/mammalian target of rapamycin (mTOR) signaling pathway under hypoxic conditions [23, 24]. Another study revealed that HVEM expressed in OVCAR3 positively regulates regulatory T cells (Tregs) by activating the STAT5/forkhead box protein P3 (Foxp3) signaling pathway in vitro [25]. However, the HVEM⁺PD‐1⁺TIM‐3⁺ cytotoxic T‐cell subpopulation was confirmed to exhibit potent antitumor effects in paclitaxel‐treated tumor‐bearing mice [26]. Because the interplay between the immune system and cancer cells in the microenvironment is orchestrated by a complex network of biological pathways, the regulatory effects of tumor cell‐expressed HVEM and the underlying mechanisms of OvCa remain largely unknown and need to be further elucidated.

In this study, we revealed the differential expression of TNFRSF14/HVEM as a tumor promoter and identified its dominant expression in CD45⁻EpCAM⁺ subsets in OvCa. Notably, in addition to promoting cancer cell proliferation, we demonstrated the immunosuppressive role of tumor cell‐expressed TNFRSF14/HVEM in accelerating OvCa progression. These findings highlight the potential of targeting TNFRSF14/HVEM as an effective therapeutic strategy for the treatment of OvCa.

## 2 Materials and Methods

### 2.1 Human Specimens

OvCa specimens (*n* = 29) and normal ovarian tissues (*n* = 7) were obtained from the Shanghai First Maternal and Infant Hospital between March 2016 and September 2023. Slices of OvCa tissues (*n* = 40) were obtained from the Department of Biobank of the Shanghai First Maternity and Infant Hospital. This study was approved by the Research Ethics Committee of the Shanghai First Maternal and Infant Hospital (Ethics code: KS22274).

### 2.2 Quantitative Real‐Time Polymerase Chain Reaction (RT‐PCR)

Total RNA was extracted using RNAiso Plus (C9109; Takara, Tokyo, Japan), and cDNA was synthesized via reverse transcription with PrimeScript™ RT Master Mix (RR036; Takara, Tokyo, Japan). Primers were synthesized by Biosue, and a QuantStudio™ 5 real‐time PCR system (Thermo Fisher & ABI, Waltham, USA) was used for quantitative PCR. Target gene mRNA expression was normalized to that of the control gene *ACTB/Actb*. All the primers used are listed in Supporting Information: Table S1.

### 2.3 Cell Culture

The murine OvCa cell line, ID8, was kindly provided by Dr. Jie Liu (Tongji University, Shanghai, China). ID8 cells were cultured in the recommended medium containing 100 U/mL penicillin, 100 U/mL streptomycin, and 10% fetal bovine serum at 37°C in a humidified 5% CO₂ atmosphere. Short tandem repeat analysis was performed to validate the cell lines. The cell line was tested every 3 months to confirm the absence of mycoplasma contamination.

### 2.4 Construction of Vectors and Transfection

The targeted short hairpin RNA was inserted into a pLKO.1 vector (Addgene #8453). 293T cells were cotransfected with the plasmids pLKO.1, pMD2.G (Addgene #12259), and psPAX2 (Addgene #12260) using Lipofectamine™ 3000 (L3000015; Thermo Fisher, Waltham, USA) to produce lentiviral particles. To generate stable knockdown cells, OvCa cells were infected with lentivirus in the presence of 8 μg/mL polybrene (H8761; Solarbio, Beijing, China). Twenty‐four hours after infection, the cells were treated with 0.6 μg/mL puromycin (HY‐B1743A; MCE, New Jersey, USA) for 3 days to eliminate uninfected cells and obtain mass populations of puromycin‐resistant cells expressing short hairpin RNAs. Western blot analysis was performed to determine knockdown efficiency. The detailed sequences of *sh‐Tnfrsf14* were as follows: Forward: CCGGGCATTTCAACAGGAAGTAAGACTCGAGTCTTACTTCCTGTTGAAATGCTTTTTG. Reverse: AATTCAAAAAGCATTTCAACAGGAAGTAAGACTCGAGTCTTACTTCCTGTTGAAATGC.

### 2.5 Cell Proliferation Assay

Cell viability was assessed using a Cell Counting Kit‐8 (CCK‐8) (C6005; NCM Biotech, Suzhou, China). A total of 1000 cells per well were seeded in 96‐well plates and incubated overnight. The culture medium was replaced with 100 μL of CCK‐8 working solution per well, and the cells were incubated at 37°C for 2 h. Cell proliferation curves were calculated by measuring absorbance at 450 nm using a microplate reader.

### 2.6 Colony Formation Assay

Cells were seeded in six‐well plates at a density of 1000 cells per well in a normal growth medium and allowed to grow for 7 days. Afterward, the cells were fixed with 4% paraformaldehyde (P0099; Beyotime, Shanghai, China) for 15 min and stained with 0.1% crystal violet (C0121; Beyotime, Shanghai, China) for 15 min at room temperature. After being washed with phosphate buffer saline (PBS), the cells were imaged, and the colonies were quantified using ImageJ software.

### 2.7 5‐Ethynyl‐2′‐deoxyUridine (EdU) Staining

We used an EdU Cell Proliferation Kit (CX003; Cellorlab, Shanghai, China) to detect and quantify cell proliferation based on EdU incorporation into newly synthesized DNA. Briefly, cells were pretreated with 10 μM EdU for 2 h at 37°C. After incubation, the cells were fixed with 4% paraformaldehyde for 15 min and permeabilized in PBS containing 0.3% Triton X‐100 (ST797; Beyotime, Shanghai, China) for 15 min at room temperature. The cells were then treated with the Click reaction buffer for 30 min at room temperature, and the nuclei were stained with Hoechst 33342 for 10 min. EdU‐positive cells were visualized under a fluorescence microscope (Nikon, Tokyo, Japan) in four random fields and quantified using ImageJ software.

### 2.8 Western Blot Analysis

Lysates from cultured cells were extracted using a total protein extraction buffer (PC101; Epizyme, Shanghai, China) containing a protease and phosphatase inhibitor cocktail (P002; NCM Biotech, Suzhou, China). Protein quantification was performed using an enhanced BCA Protein Assay Kit (P0010; Beyotime, Shanghai, China) according to the manufacturer's instructions. Equal amounts of protein were electrophoresed on 10% sodium dodecyl sulfate‐polyacrylamide gels and transferred to nitrocellulose membranes (P‐66485; PALL, Port Washington, USA). The membranes were blocked with 5% nonfat milk at room temperature for 1 h, followed by overnight incubation with appropriate concentrations of primary antibodies. The blots were further incubated with a horseradish peroxidase‐conjugated anti‐rabbit secondary antibody (LF102; Epizyme, Shanghai, China) for 1 h at room temperature, and the bands were visualized using a Tanon 4600 Chemiluminescent Imaging System (Tanon, Shanghai, China). The primary antibodies used were as follows: HVEM (10138‐1‐AP; Proteintech, Chicago, USA), XBP1 (220783; Abcam, Cambridge, UK), c‐Myc (18583; CST, Danvers, USA), phospho‐NF‐κB p65‐Ser536 (3033T; CST, Danvers, USA), phospho‐STAT1‐Y701 (AP0135; ABclonal, Woburn, USA), phospho‐STAT5‐Y694 (AP0887; ABclonal, Woburn, USA), phospho‐STAT6‐Y641 (ab235591; Abcam, Cambridge, UK), and β‐Actin (AC026; ABclonal, Woburn, USA).

### 2.9 Mice

C57BL/6 mice were purchased from Beijing Vital Star Biotechnology Co. Ltd. All mice were bred and maintained under specific pathogen‐free conditions. The mice were fed a standard diet, had free access to food and water, and were housed in a 12‐h light‐dark cycle. Female mice, 6 weeks old, were used for the experiments in this study. All procedures were approved by the Institutional Animal Care and Use Committee of Tongji University (Ethics code: TJBG00324101).

### 2.10 OvCa Model

The mice were intraperitoneally injected with 5 × 10⁶ *Ctrl‐*ID8 or *Tnfrsf14* ^*KD*^‐ID8 cells for the intraperitoneal model. Seven weeks after injection, the mice were killed, and ascites and spleens were harvested for flow cytometry analysis. The number of nodules on the abdominal walls of the mice was calculated.

### 2.11 Cell Isolation

Fresh OvCa specimens were washed with precooled PBS, minced into small pieces, and then incubated with 1 mg/mL type IV collagenase (C5138; Sigma‐Aldrich, Wisconsin, USA) and 0.1 mg/mL DNase I (DN25; Sigma‐Aldrich, Wisconsin, USA) at 37°C, 220 rpm for 45 min in a constant‐temperature shaker. The suspension was passed through a 70‐μm nylon cell strainer. The cells were collected by centrifugation at 1500 rpm for 5 min and resuspended in fluorescence‐activated cell sorting (FACS) staining buffer for cell marker staining.

The spleen was excised and homogenized using frosted microscope slides. The cells were treated with RBC lysis buffer (420301; BioLegend, San Diego, CA, USA) for 5 min at room temperature, collected by centrifugation at 1500 rpm for 5 min, and resuspended in FACS staining buffer for cell marker staining.

Ascites were collected using a sterile syringe and centrifuged at 1500 rpm for 5 min. The supernatant was removed, and the cells were treated with RBC lysis buffer for 5 min at room temperature, centrifuged, and resuspended in FACS staining buffer for cell marker staining.

### 2.12 Flow Cytometry and Antibodies

For cell surface marker analysis, cells were preincubated with a purified CD16/32 antibody (553141; BD Biosciences, New Jersey, USA) and then stained with fluorescent‐labeled antibodies in FACS staining buffer for 30 min on ice in the dark. The following antibodies were used in this study and purchased from BioLegend (San Diego, USA): anti‐mouse CD45 (103133), anti‐mouse CD11b (101206), anti‐mouse F4/80 (123114), anti‐mouse CD11c (117309), anti‐mouse Gr‐1 (108411), anti‐mouse CD3 (100204), anti‐mouse CD4 (100434), anti‐mouse CD8a (100712), anti‐mouse CD19 (115519), anti‐mouse NK‐1.1 (108707), anti‐mouse CD44 (103007), anti‐mouse CD62L (104417), anti‐human CD45 (304008), anti‐human CD326 (369810), and anti‐human CD270 (318810).

Cells were activated using a Cell Activation Cocktail (with Brefeldin A) (423304; BioLegend, San Diego, USA) for 5 h to promote cytokine production. For intracellular staining, cells underwent surface staining, were immediately fixed with BD Cytofix/Cytoperm™ Fixation and Permeabilization Solution (554722; BD Biosciences, New Jersey, USA) for 20 min on ice in the dark, and then incubated with intracellular antibodies for 30 min on ice. The following antibodies were utilized: anti‐mouse IFN‐γ (505806; BioLegend, San Diego, USA), anti‐mouse IL‐4 (504103; BioLegend, San Diego, USA), and anti‐mouse IL‐17A (506915; BioLegend, San Diego, USA).

For Foxp3 staining, cells were incubated with anti‐mouse CD4 (100434; BioLegend, San Diego, USA) and anti‐mouse CD25 (101907; BioLegend, San Diego, USA) for 30 min on ice in the dark, immediately fixed with the Foxp3/Transcription Factor Staining Buffer Set (00‐5523‐00; Thermo Fisher & Invitrogen, Waltham, USA) for 30 min on ice in the dark, and then incubated with anti‐mouse FOXP3 (126403; BioLegend, San Diego, USA) for 30 min on ice.

Data were acquired using a BD Canto II instrument (BD Biosciences, New Jersey, USA) and analyzed using FlowJo software.

### 2.13 Immunofluorescent Staining

Slices were obtained from the Department of Biobank of Shanghai First Maternity and Infant Hospital. This assay was supported by Ningbo Yangming Medical Inspection Laboratory Co. Ltd. (Ningbo, China). TNFRSF14 Rabbit Monoclonal Antibody (PSH06‐02) and EpCAM Rabbit Monoclonal Antibody (PS01‐69) were purchased from Huabio (Hangzhou, China).

### 2.14 Data Set Information and Bioinformatics Analysis

The RNA expression of 379 patients with OvCa in the TCGA cohort was downloaded from the UCSC Xena database (https://gdc.xenahubs.net, ID: TCGA‐OV.htseq_fpkm, TCGA‐OV.GDC_phenotype). To better understand the biological role of HVEM, OvCa samples were divided into HVEM^high^ and HVEM^low^ subgroups based on the median expression of HVEM. Differentially expressed genes (DEGs) were analyzed using the “limma” package [27] between those with high and low HVEM expression in the TCGA datasets, with significance set at adj. *p*‐value < 0.001. The “gseaplot” package [28] was used for gene set enrichment analysis, with selection criteria including FDR < 0.05 and |NES | > 1. The expression of genes ranked tenth based on |logFC| was analyzed in the chemokine and cytokine pathways between the two subgroups.

### 2.15 Statistical Analysis

Statistical analysis was conducted using GraphPad Prism 10 software. All data were presented as mean ± standard deviation (SD). Two‐tailed Student's *t*‐tests were used to compare two independent groups. An unpaired *t*‐test was used to compare observations between two study groups when the groups were independent. For all analyses, *p* < 0.05 was considered statistically significant.

## 3 Results

### 3.1 TNFRSF14/HVEM Was Overexpressed in OvCa

**Figure 1:**
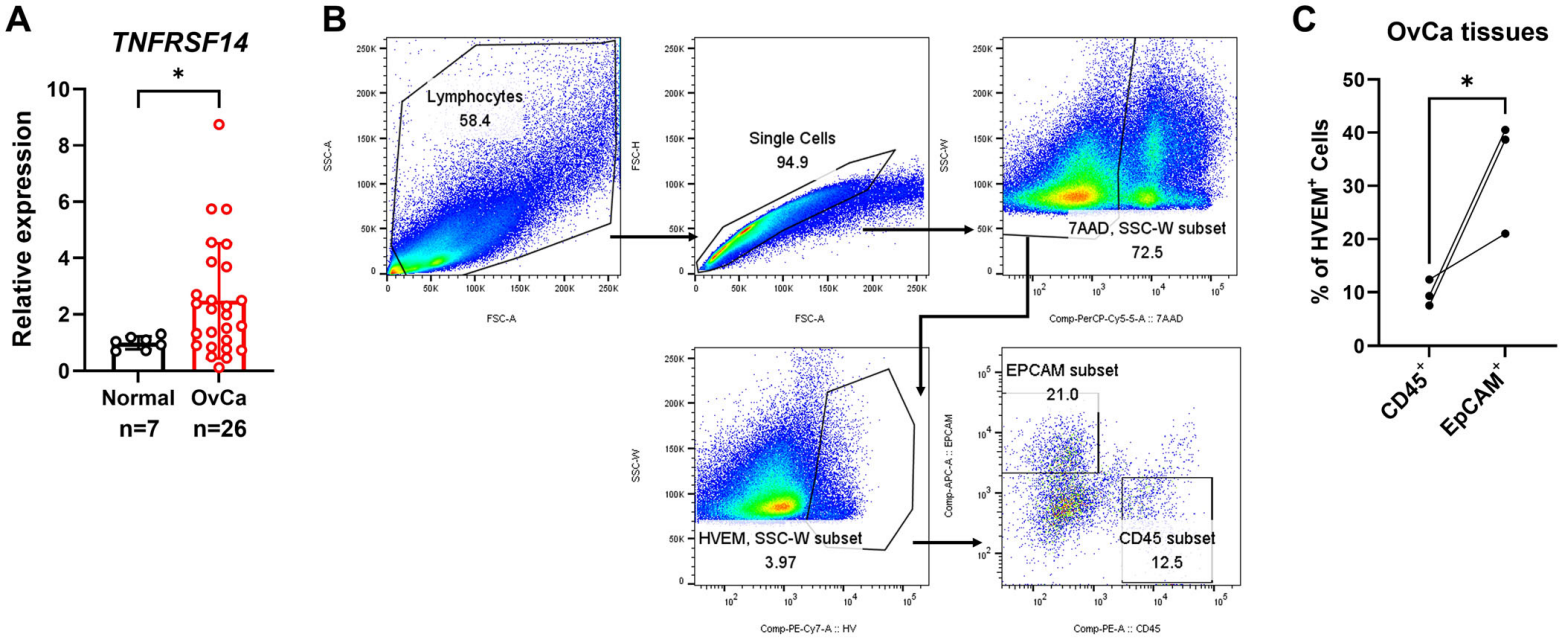
The expression of TNFRSF14/HVEM in surgical resection specimens in OvCa. (A) The mRNA expression level of *TNFRSF14* in specimens from patients with benign tumors (*n* = 7) and OvCa (*n* = 26) was detected by quantitative RT‐PCR. (B) Flow cytometry gating strategy for HVEM expression in different cell subsets in specimens from OvCa patients. (C) The ratio of HVEM‐positive cells in CD45⁺EpCAM^−^ and CD45^−^EpCAM⁺ subsets in specimens from OvCa patients (*n* = 3) was detected by flow cytometry. Data were presented as means ± SD. Two‐tailed unpaired Student's *t*‐test in (A) and (C). *p < 0.05, **p < 0.01, ***p < 0.001; ns, no significance. HVEM, herpesvirus entry mediator; OvCa, ovarian cancer; RT‐PCR, real‐time polymerase chain reaction; SD, standard deviation; TNFRSF14, tumor necrosis factor superfamily member 14.

To identify potential TNFRSF14/HVEM dysregulation in OvCa, we first analyzed the mRNA expression level of *TNFRSF14* in specimens from patients with benign tumors (*n* = 7) and patients with OvCa (*n* = 26). The data showed that *TNFRSF14* expression in ovarian tissues from patients with OvCa was markedly higher than that in benign tissues (Figure 1A). The relation between the ratio of *TNFRSF14/ACTB* mRNA expression and clinicopathological parameters in OvCa tissues is summarized in Table 1. Cellular components in the tumor microenvironment of OvCa include cancer cells, cancer‐associated fibroblasts, endothelial cells, immune cells, and others [29]. Subsequently, we aimed to identify the dominant cell type in which HVEM expression was elevated. We prepared a single‐cell suspension of four OvCa tissues and detected HVEM expression in cancer cells (CD45^−^EpCAM⁺) and immune cells (CD45⁺EpCAM^−^) by flow cytometry (the gating strategy is shown in Figure 1B). Consequently, the HVEM expression of CD45^−^EpCAM⁺ cells was higher than that of CD45⁺EpCAM^−^ cells in three‐quarters of the OvCa tissues. The remaining specimen was excluded from the graph (Figure 1C). These results demonstrate that TNFRSF14/HVEM is overexpressed in OvCa, especially in CD45^−^EpCAM⁺ cancer cells.

**Table 1:** The relationship between the ratio of *TNFRSF14/ACTB* mRNA expression and clinicopathological parameters in ovarian cancer tissues.

Clinical features	Sample size	Expression	*t*‐value	*p*‐value
Age				
≤ 50	6	2.264 ± 0.552	0.388	0.704
> 50	20	2.552 ± 0.498		
Lymphatic metastasis				
No	16	2.419 ± 0.560	0.080	0.938
Yes	5	2.343 ± 0.764		
Vascular infiltration				
No	21	2.519 ± 0.468	0.197	0.850
Yes	5	2.343 ± 0.764		
FIGO stage				
I–II	14	2.884 ± 0.646	0.420	0.679
III	8	2.546 ± 0.481		

Abbreviations: FIGO, International Federation of Gynecology and Obstetrics; TNFRSF14, tumor necrosis factor superfamily member 14.

### 3.2 Knockdown of *Tnfrsf14* Inhibited OvCa Cell Proliferation

**Figure 2:**
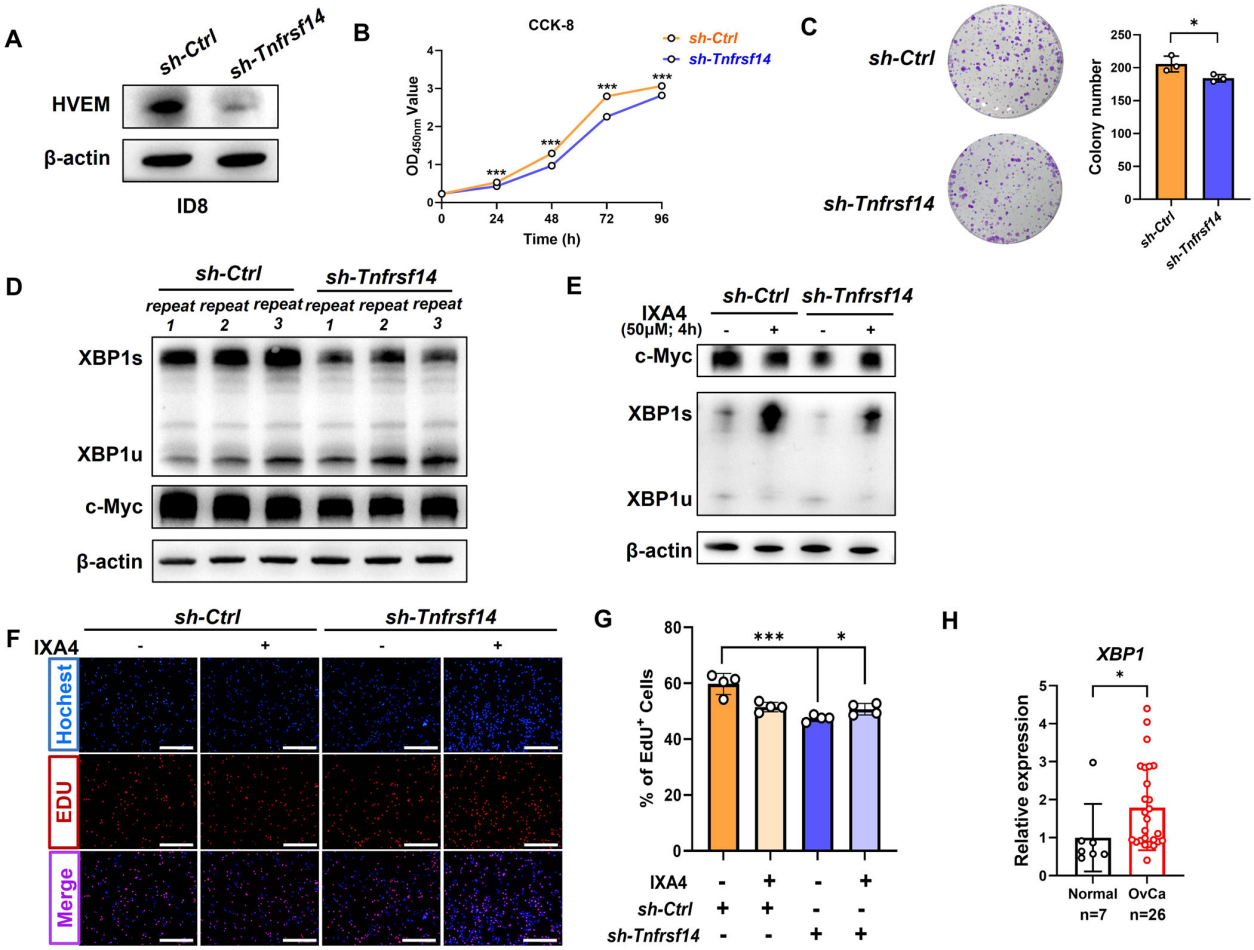
Knockdown of *Tnfrsf14* inhibited OvCa cell proliferation. (A) The murine OvCa cell line ID8 was infected with lentivirus to generate stable *Tnfrsf14* ^KD^ cells. The shRNA‐mediated *Tnfrsf14* knockdown efficiency was detected by western blot analysis. *Ctrl*‐ID8 cells: *sh*‐*Ctrl*, *Tnfrsf14* ^KD^‐ID8 cells: *sh*‐*Tnfrsf14*. (B) The CCK−8 assay was used to determine the difference in cell viability between *Ctrl*‐ID8 and *Tnfrsf14* ^KD^‐ID8 cells. (C) The colony formation assay was used to detect the proliferation ability of *Ctrl*‐ID8 and *Tnfrsf14* ^KD^‐ID8 cells. The representative digital images (left) and statistical analysis (right) were shown. (D) The protein levels of XBP1s (the spliced form of XBP1), XBP1u (the unspliced form of XBP1), and c‐Myc in *Ctrl*‐ID8 and *Tnfrsf14* ^KD^‐ID8 cells were performed by western blot analysis. (E) The *Ctrl*‐ID8 and *Tnfrsf14* ^KD^‐ID8 cells were treated with 50 μΜ IXA4 or equal volumes of DMSO for 4 h and the protein levels of XBP1s, XBP1u, and c‐Myc were performed by western blot analysis. (F) The *Ctrl*‐ID8 and *Tnfrsf14* ^KD^‐ID8 cells were plated and treated with 50 μΜ IXA4 or equal volumes of DMSO for 4 h. The EdU assay was used to detect the difference in cell proliferation among each group. The representative digital images were shown. Scale bar: 200 μm. (G) The statistical analysis of the ratio of EdU‐positive cells among each group was shown. (H) The mRNA expression level of *XBP1* in specimens from patients with benign tumors (*n* = 7) and OvCa (*n* = 26) was detected by quantitative RT‐PCR. Data were presented as means ± SD. Two‐tailed unpaired Student's *t*‐test in (B), (C), (G), and (H). *p < 0.05, **p < 0.01, ***p < 0.001. CCK‐8, Cell Counting Kit‐8; DMSO, dimethyl sulfoxide; EdU, 5‐ethynyl‐2′‐deoxyuridine; ns, no significance; OvCa, ovarian cancer; RT‐PCR, real‐time polymerase chain reaction; SD, standard deviation; shRNA, short hairpin RNA; TNFRSF14, tumor necrosis factor superfamily member 14.

To elucidate the biological functions of TNFRSF14/HVEM in OvCa, we first generated stable *Tnfrsf14* ^KD^‐ID8 cells and confirmed their knockdown efficiency using western blot analysis. The results showed that the stable expression of short hairpin RNA targeting *Tnfrsf14* resulted in an obvious decrease in HVEM protein expression in murine ID8 cells (Figure 2A). A fundamental characteristic of cancer cells is their ability to sustain persistent proliferation [30]. To investigate the possible function of *Tnfrsf14* in cancer cells, a CCK‐8 assay was performed to evaluate cell viability. Our data revealed that decreased expression of TNFRSF14/HVEM in ID8 cells significantly inhibited cancer cell growth (Figure 2B). Similarly, a reduced proportion of EdU‐positive cells was observed by EdU staining in *Tnfrsf14* ^KD^‐ID8 cells compared with that in *Ctrl*‐ID8 cells, indicating suppressed cancer cell proliferation (Figure 2C).

XBP1 plays an important role in the unfolded protein response in the endoplasmic reticulum (ER), and its function in various tumors is increasingly recognized [31]. Activated inositol‐requiring enzyme 1 (IRE1), an ER sensor protein, increases RNase activity and excises a 26‐nucleotide intron (531–556) from unspliced XBP1 (XBP1u) mRNA to produce spliced XBP1 (XBP1s) [32]. The regulatory activity of XBP1 in promoting cancer cell proliferation has been well‐reviewed [33]. c‐Myc is a direct downstream target of XBP1, regulating proliferation [32]. To investigate the molecular mechanism by which TNFRSF14/HVEM promotes cell proliferation in OvCa, we analyzed the protein levels of XBP1s, XBP1u, and c‐Myc in *Ctrl*‐ID8 and *Tnfrsf14* ^KD^‐ID8 cells. We observed that *Tnfrsf14* knockdown significantly decreased XBP1s and c‐Myc expression (Figure 2D). IXA4, a highly selective and nontoxic IRE1/XBP1s activator, selectively upregulates the mRNA level of XBP1s [34]. Treatment with IXA4 partially rescued the decrease in XBP1s and c‐Myc expression induced by *Tnfrsf14* knockdown in ID8 cells (Figure 2E). Moreover, the reduced proportion of EdU‐positive cells in *Tnfrsf14* ^KD^‐ID8 cells indicated suppressed cell proliferation compared with that in *Ctrl*‐ID8 cells, which was restored by IXA4 treatment (Figure 2F,G). In clinical specimens, *XBP1* expression in ovarian tissues from patients with OvCa was remarkably higher than that in ovarian tissues from patients with benign tumors (Figure 2H). The relation between the ratio of *XBP1/ACTB* mRNA expression and clinicopathological parameters in OvCa tissues is summarized in Table 2. Collectively, these results suggest that TNFRSF14/HVEM contributes to OvCa proliferation.

**Table 2:** The relationship between the ratio of *XBP1/ACTB* mRNA expression and clinicopathological parameters in ovarian cancer tissues.

Clinical features	Sample size	Expression	*t*‐value	*p*‐value
Age				
≤ 50	6	1.988 ± 0.564	0.422	0.686
> 50	20	1.729 ± 0.237		
Lymphatic metastasis				
No	16	1.781 ± 0.269	0.648	0.545
Yes	5	2.284 ± 0.729		
Vascular infiltration				
No	21	1.671 ± 0.213	1.106	0.280
Yes	5	2.284 ± 0.729		
FIGO stage				
I–II	14	1.920 ± 0.298	0.994	0.334
III	8	1.480 ± 0.326		

Abbreviation: FIGO, International Federation of Gynecology and Obstetrics.

### 3.3 Knockdown of *Tnfrsf14* Alleviated Ovca Progression in Mice

**Figure 3:**
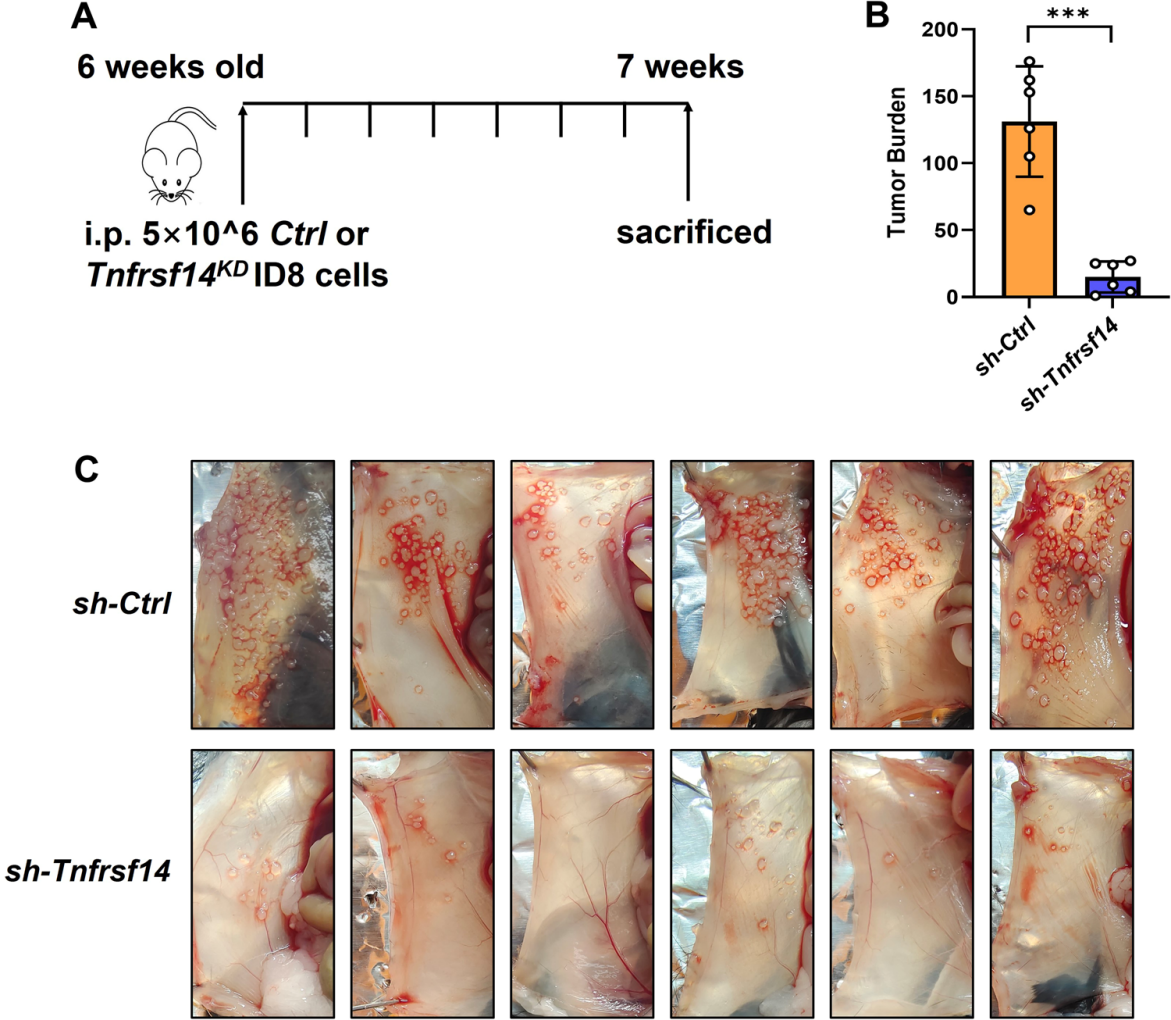
Knockdown of *Tnfrsf14* alleviated OvCa progression in mice. (A) The schematic diagram illustrating the intraperitoneal OvCa model in female C57BL/6 mice. (B) The statistical analysis for the number of nodules on the abdominal walls of mice (*n* = 6). (C) The representative digital images for the number of nodules on the abdominal walls of mice (*n* = 6). Data were presented as means ± SD. Two‐tailed unpaired Student's *t*‐test in (B). *p < 0.05; **p < 0.01; ***p < 0.001. ns, no significance; OvCa, ovarian cancer; SD, standard deviation.

We examined the effect of TNFRSF14/HVEM in mice. Mice were intraperitoneally injected with 5 × 10⁶ *Ctrl*‐ID8 or *Tnfrsf14* ^KD^‐ID8 cells in an intraperitoneal model and killed 7 weeks later (Figure 3A). Compared to *Ctrl*‐ID8 tumor‐bearing mice, the number of nodules on the abdominal walls of *Tnfrsf14* ^KD^‐ID8 tumor‐bearing mice was significantly lower (Figure 3B,C). These data suggest that TNFRSF14/HVEM efficiently promotes the progression of OvCa.

### 3.4 Knockdown of *Tnfrsf14* Altered the Frequency of Lymphoid Cells in OvCa Mice

**Figure 4:**
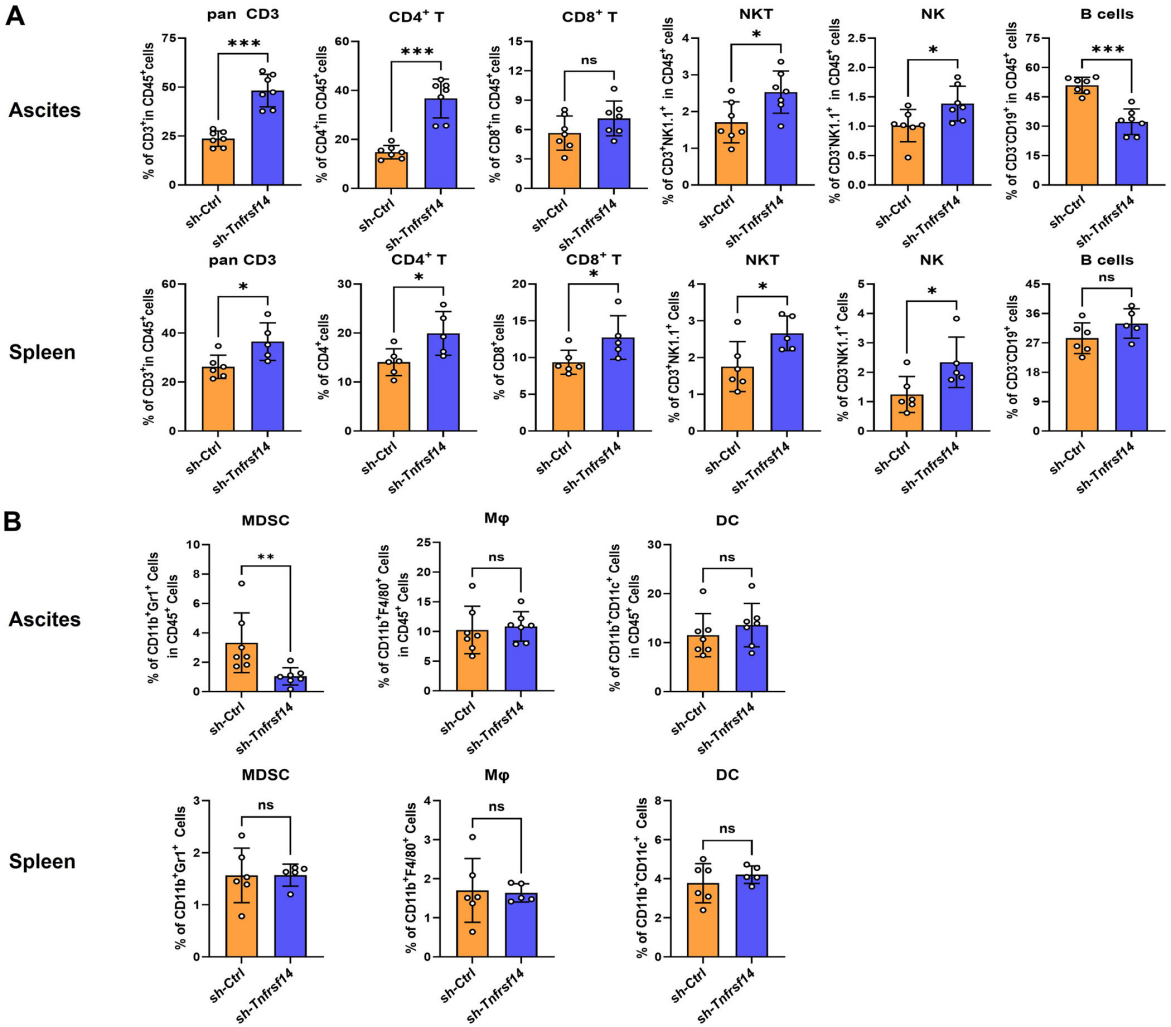
Knockdown of *Tnfrsf14* altered the frequency of lymphoid cells in OvCa mice. The statistical analysis for the frequency of lymphoid cells (A) and myeloid cells (B) in the ascites and spleen of mice was shown. Ascites: mice injected with *Ctrl*‐ID8 cells (*n* = 7); mice injected with *Tnfrsf14* ^KD^‐ID8 cells (*n* = 7). Spleen: mice injected with *Ctrl*‐ID8 cells (*n* = 6); mice injected with *Tnfrsf14* ^KD^‐ID8 cells (*n* = 5). Data were presented as means ± SD. Two‐tailed unpaired Student's *t*‐test in (A) and (B). *p < 0.05; **p < 0.01; ***p < 0.001. ns, no significance; OvCa, ovarian cancer; SD, standard deviation.

To delineate the potential immune constituents within the tumor microenvironment influenced by TNFRSF14/HVEM, we investigated both lymphoid cells (CD3⁺ T cells, CD4⁺ T cells, CD8⁺ T cells, natural killer T cells [NKTs], natural killer cells [NKs], and B cells) and myeloid cells (myeloid‐derived suppressor cells [MDSCs], macrophages, and dendritic cells) in the ascites and spleens of both *Ctrl*‐ID8 tumor‐bearing mice and *Tnfrsf14* ^KD^‐ID8 tumor‐bearing mice. Intriguingly, we observed a specific increase in the frequency of CD3⁺ T cells, CD4⁺ T cells, NKTs, and NKs in the ascites of *Tnfrsf14* ^KD^‐ID8 tumor‐bearing mice. Despite no statistical difference, an upward trend was observed in the frequency of CD8⁺ T cells in the ascites of *Tnfrsf14* ^KD^‐ID8 tumor‐bearing mice. However, the frequency of B cells in the ascites was low in *Tnfrsf14* ^KD^‐ID8 tumor‐bearing mice (Figure 4A). We also observed an increase in splenic CD3⁺ T cells, CD4⁺ T cells, CD8⁺ T cells, NKTs, and NKs in *Tnfrsf14* ^KD^‐ID8 tumor‐bearing mice. The frequency of splenic B cells was comparable between the two groups of ID8 tumor‐bearing mice (Figure 4A). By contrast, no obvious discrepancy was found in the myeloid cells mentioned in the ascites and spleens between the two groups of ID8 tumor‐bearing mice, except for ascitic MDSCs. There was a significant decrease in the frequency of ascitic MDSCs in *Tnfrsf14* ^KD^‐ID8 tumor‐bearing mice compared to that in *Ctrl*‐ID8 tumor‐bearing mice (Figure 4B). Additionally, we investigated CD3⁺ T‐cell infiltration in specimens from patients with OvCa (*n* = 40) using immunofluorescence staining. As expected, specimens with low HVEM expression in epithelial tumor cells exhibited significant CD3⁺ T‐cell infiltration (Supporting Information: Figure S1). Thus, TNFRSF14/HVEM specifically altered the frequency of lymphoid cells in the tumor microenvironment of OvCa mice.

### 3.5 Knockdown of *Tnfrsf14* Altered the Function of T Cells in OvCa Mice

**Figure 5:**
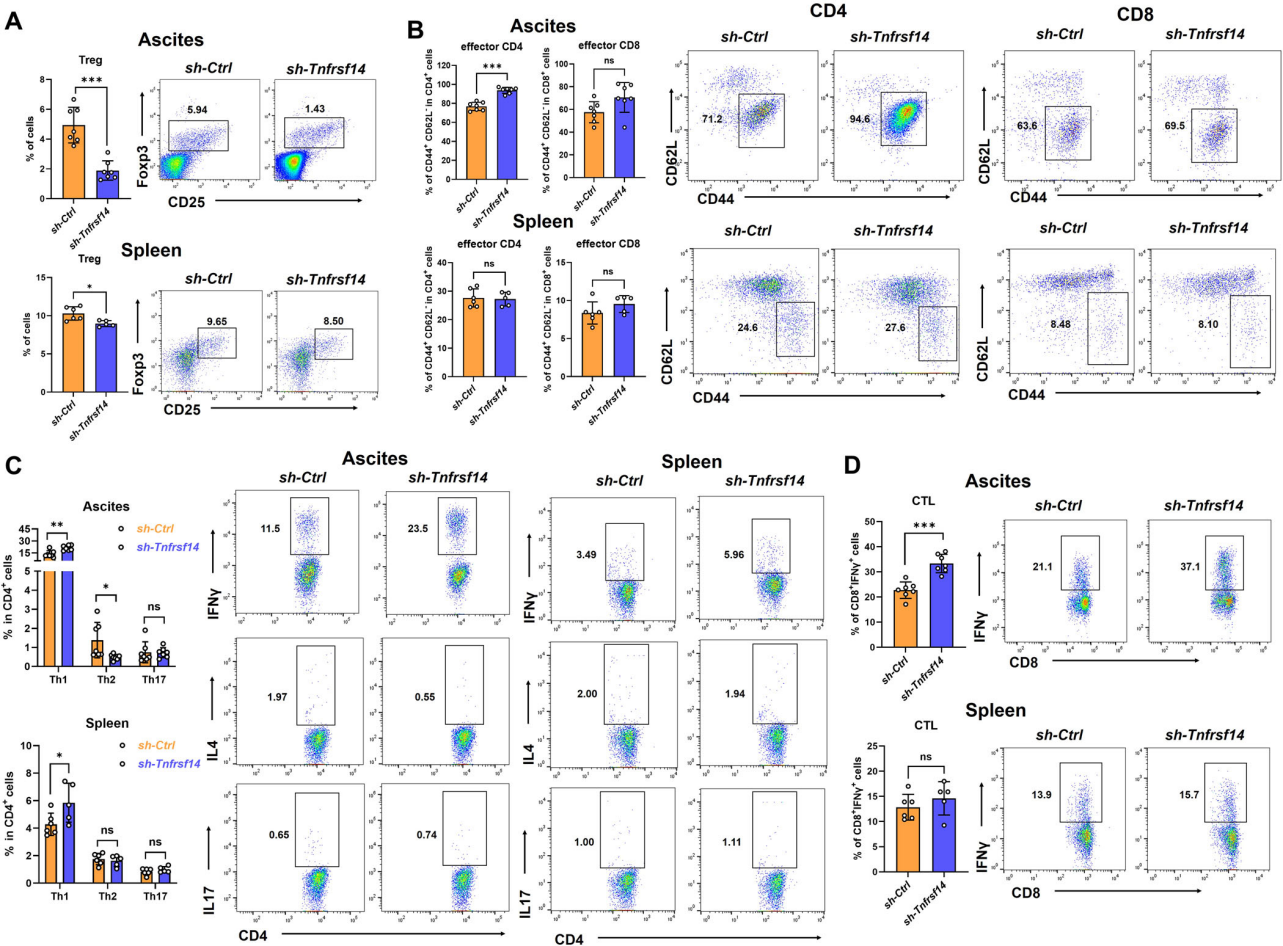
Knockdown of *Tnfrsf14* altered the function of T cells in OvCa mice. (A) The frequency (left) and representative flow cytometry scatter plots (right) of CD4^+^CD25^+^Foxp3^+^ regulatory T cells in the ascites and spleen of mice. (B) The frequency (left) of CD4^+^CD44^+^CD62L^−^ effector T cells and CD8^+^CD44^+^CD62L^−^ effector T cells in the ascites and spleen of mice was analyzed. The representative flow cytometry scatter plots of CD4^+^ cells (middle) and CD8^+^ cells (right) were shown. (C) The IFNγ, IL4, and IL17 expression patterns in the CD4^+^ T cells in the ascites and spleen of mice were summarized (left) and the representative flow cytometry scatter plots were shown (right). (D) The IFNγ expression patterns in the CD8^+^ T cells in the ascites and spleen of mice were summarized (left) and the representative flow cytometry scatter plots were shown (right). Ascites: mice injected with *Ctrl*‐ID8 cells (*n* = 7); mice injected with *Tnfrsf14* ^KD^‐ID8 cells (*n* = 7). Spleen: mice injected with *Ctrl*‐ID8 cells (*n* = 6); mice injected with *Tnfrsf14* ^KD^‐ID8 cells (*n* = 5). Data were presented as means ± SD. Two‐tailed unpaired Student's *t*‐test in (A–D). *p < 0.05; **p < 0.01; ***p < 0.001. IFNγ, interferon‐gamma; IL, interleukin; ns, no significance; OvCa, ovarian cancer; SD, standard deviation.

We evaluated the function of T cells within the tumor microenvironment influenced by TNFRSF14/HVEM. Tregs, commonly known as CD4⁺CD25⁺Foxp3⁺, make up approximately 5% of the T‐cell population and diminish antitumor immunity by allowing cancer cells to evade the antitumor response [35]. There was a lower frequency of Tregs in both the ascites and spleens of *Tnfrsf14* ^KD^‐ID8 tumor‐bearing mice compared to those in the *Ctrl*‐ID8 tumor‐bearing mice (Figure 5A). The effector memory T cell (T_EM_), characterized as CD44^highCD62L⁻ [36], exerts rapid effector function in antitumor immunity [37]. The results revealed a significant increase in the frequency of ascitic T_EM_ in CD4⁺ T cells but not in CD8⁺ T cells in *Tnfrsf14* ^KD^‐ID8 tumor‐bearing mice. Unexpectedly, we failed to observe any changes in splenic CD4⁺ T_EM_ or CD8⁺ T_EM_ between the two groups of mice (Figure 5B). In ascitic CD4⁺ T cells of tumor‐bearing mice, *Tnfrsf14* knockdown resulted in a reciprocal increase in IFNγ‐producing Th1 cells and a decrease in IL4‐producing Th2 cells, whereas the levels of IL17‐producing Th17 cells remained similar. An increase in IFNγ‐producing Th1 cells was also observed in splenic CD4⁺ T cells (Figure 5C). Although *Tnfrsf14* knockdown in ID8 cells did not alter the frequency of CD8⁺ T cells or CD8⁺ T_EM_ in the ascites, the robust elevation in the number of cytotoxic T lymphocytes (CTLs, IFNγ‐producing CD8⁺ T cells) in *Tnfrsf14* ^KD^‐ID8 tumor‐bearing mice indicates enhanced antitumor immunity (Figure 5D). In stark contrast to ascites, *Tnfrsf14* knockdown in ID8 cells did not alter the frequency of splenic CTLs or CD8⁺ T_EM_, despite an increase in total CD8⁺ T cells (Figure 5D). Collectively, these data demonstrate that TNFRSF14/HVEM predominantly inhibits antitumor immunity in the ascites of OvCa mice and exhibits limited effects on peripheral immunity.

### 3.6 Knockdown of *Tnfrsf14* Altered the Expression of Cytokines and Chemokines in ID8 Cells

**Figure 6:**
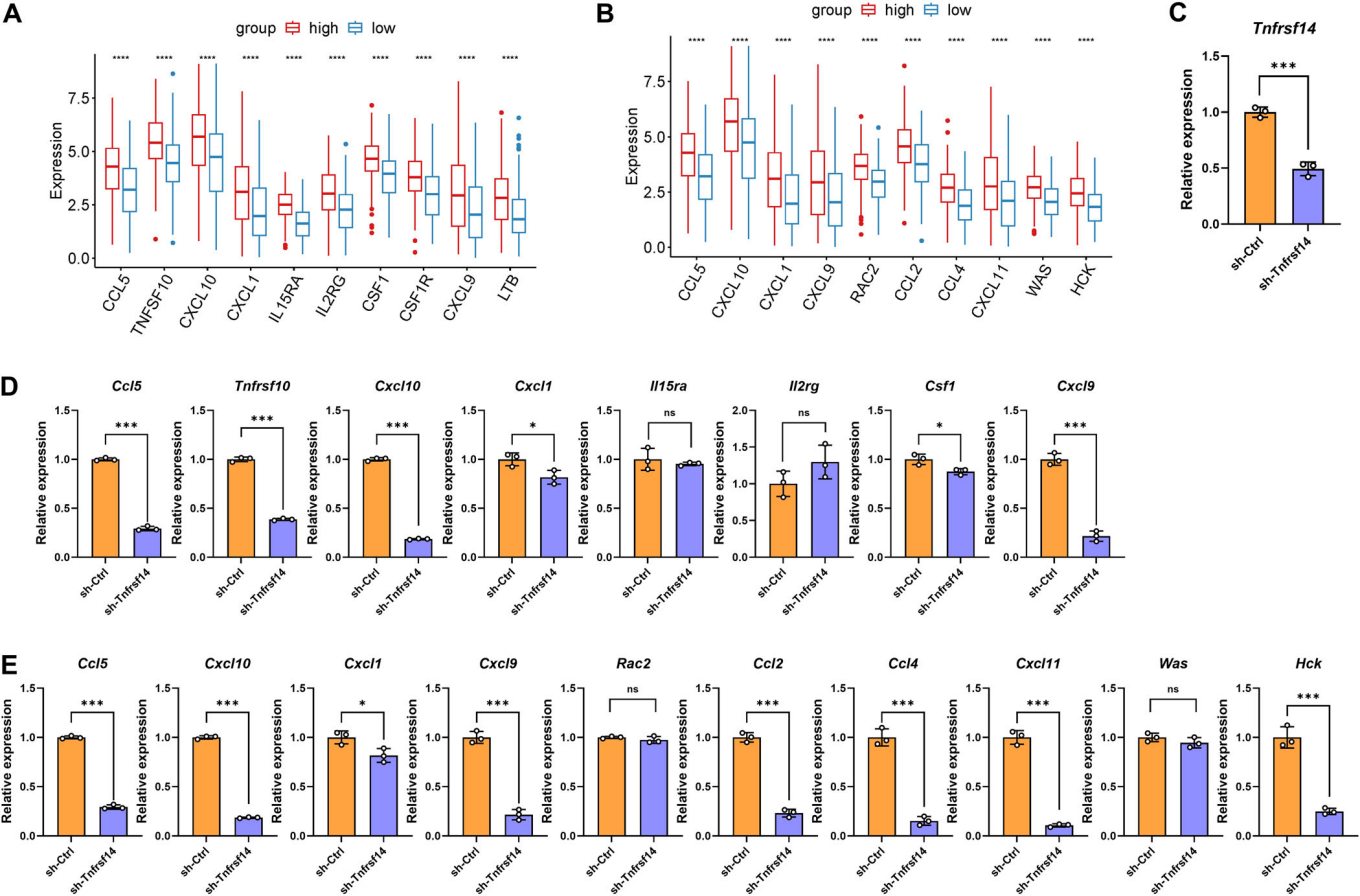
Knockdown of *Tnfrsf14* altered the expression of cytokines and chemokines in ID8 cells. (A, B) We obtained gene expression data from 379 OvCa samples in the TCGA database. These samples were divided into a high‐expression group and a low‐expression group according to the median expression level of the *TNFRSF14* gene, and the differently expressed genes (DEGs) were analyzed using the limma package. These DEGs with the top 10 |logFC| (fold change) in the cytokine (A) and chemokine (B) pathways were displayed in the histogram. (C) The mRNA expression level of *Tnfrsf14* in *Ctrl*‐ID8 and *Tnfrsf14* ^KD^‐ID8 cells was detected by quantitative RT‐PCR. (D, E) The mRNA expression levels of the top 10 cytokines (D) and chemokines (E) in *Ctrl*‐ID8 and *Tnfrsf14* ^KD^‐ID8 cells were detected by quantitative RT‐PCR. The identical statistical plots for *Ccl5*, *Cxcl10*, *Cxcl1*, and *Cxcl9* were shown twice in (D) and (E) as a match for (A) and (B). Data were presented as means ± SD. Two‐tailed unpaired Student's *t*‐test in (C–E). *p < 0.05; **p < 0.01; ***p < 0.001. ns, no significance; OvCa, ovarian cancer; RT‐PCR, real‐time polymerase chain reaction; SD, standard deviation.

To date, more than 300 cytokines, chemokines (chemoattractant cytokines), and growth factors have been extensively investigated, demonstrating distinct pro‐ and antitumorigenic effects [38, 39]. To identify the cytokines and chemokines regulated by TNFRSF14/HVEM, 379 OvCa samples from the TCGA cohort were divided into a high‐expression group and a low‐expression group according to the median expression level of the *TNFRSF14* gene. The top 10 genes based on |logFC| in the chemokine and cytokine pathways between the two subgroups were identified (Figure 6A,B). Subsequently, we tested the mRNA expression in *Tnfrsf14* ^KD^‐ID8 and *Ctrl*‐ID8 cells using quantitative RT‐PCR (Figure 6C). Among the top 10 cytokines, knockdown of *Tnfrsf14* in ID8 cells significantly suppressed *Ccl5*, *Tnfrsf10*, *Cxcl10*, *Cxcl1*, *Csf1*, and *Cxcl9* expression. There was no discrepancy in *Il15ra* and *Il2rg* expression between *Tnfrsf14* ^KD^‐ID8 cells and *Ctrl*‐ID8 cells. Unexpectedly, the remaining two genes (*Csf1r* and *Ltb*) were not detected in ID8 cells (Figure 6D). This may be because these genes are expressed in other cell constituents of OvCa tissues rather than in epithelial cells. Among the top 10 chemokines, knockdown of *Tnfrsf14* in ID8 cells significantly suppressed *Ccl5*, *Cxcl10*, *Cxcl1*, *Cxcl9*, *Ccl2*, *Ccl4*, *Cxcl11*, and *Hck* expression. There was no difference in *Rac2* and *Was* expression between *Tnfrsf14* ^KD^‐ID8 and *Ctrl*‐ID8 cells (Figure 6E). These data suggest that TNFRSF14/HVEM expressed on tumor cells exerts immunoregulatory effects by altering the expression of cytokines and chemokines.

### 3.7 Knockdown of *Tnfrsf14* Altered the Activation of the STAT Signal in ID8 Cells

**Figure 7:**
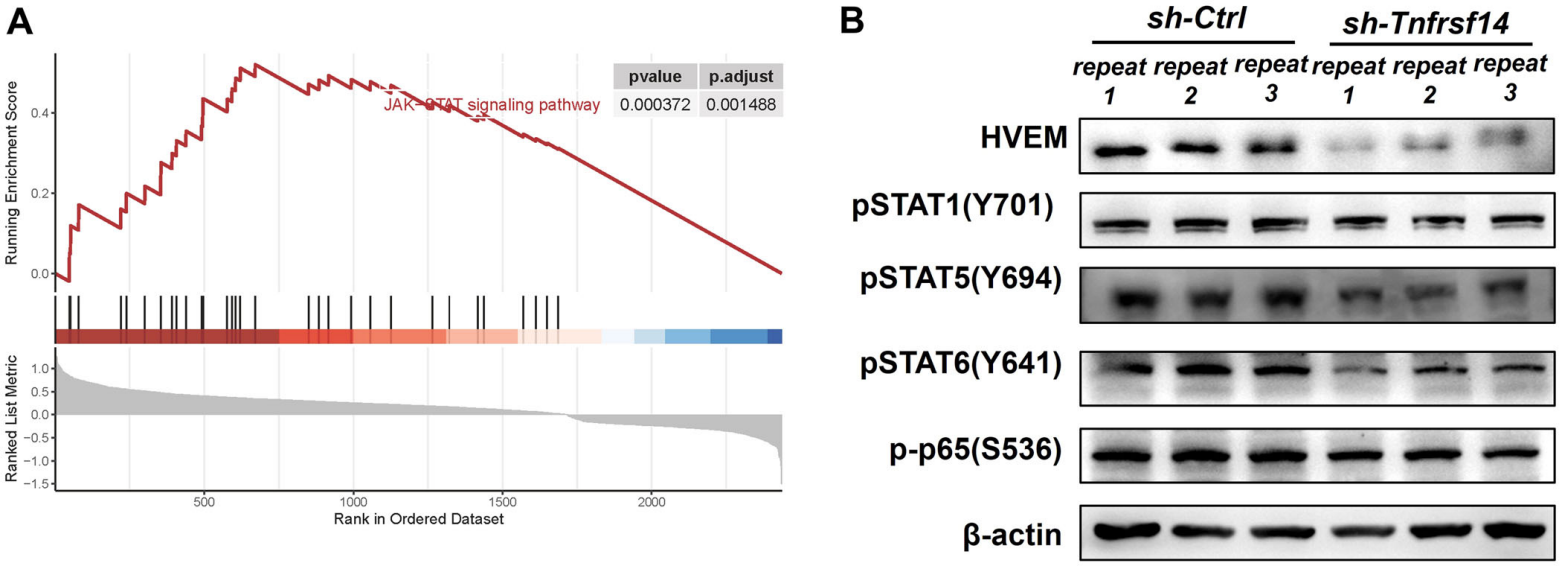
Knockdown of *Tnfrsf14* altered the activation of the STAT signal in ID8 cells. (A) We obtained gene expression data from 379 patients with OvCa in the TCGA database. OvCa samples were divided into HVEM^high^ and HVEM^low^ subgroups according to the median expression level of *TNFRSF14* gene. The gene set enrichment analysis plot was based on the gene expression profiles of HVEM^high^ and HVEM^low^ subgroups. (B) The protein levels of phospho‐Stat1 (Tyr701), phospho‐Stat5 (Tyr694), phospho‐Stat6 (Tyr641), and phospho‐NF‐κB p65‐Ser536 in *Ctrl*‐ID8 and *Tnfrsf14* ^KD^‐ID8 cells were performed by western blot analysis. HVEM, herpesvirus entry mediator; OvCa, ovarian cancer; TNFRSF14, tumor necrosis factor receptor superfamily member 14.

We then explored the signaling pathways affected by TNFRSF14/HVEM. The gene set enrichment analysis plot was based on the gene expression profiles of the *TNFRSF14*‐high‐expression group and *TNFRSF14*‐low‐expression group. The JAK‐STAT signaling pathway was upregulated in the *TNFRSF14*‐high‐expression group (Figure 7A). Western blot analysis verified the downregulation of phospho‐Stat5 (Tyr694) and phospho‐Stat6 (Tyr641) in *Tnfrsf14* ^KD^‐ID8 cells compared with that in *Ctrl*‐ID8 cells. No differences were observed in the expression of phospho‐Stat1 (Tyr701) between the two cell lines (Figure 7B). The JAK‐STAT signal can interact with nuclear factor‐κB (NF‐κB), and constitutively activated NF‐κB in cancers is usually considered pivotal for many protumorigenic functions [40]. Unfortunately, we failed to observe any obvious changes in phospho‐NF‐κB p65‐Ser536 expression in *Tnfrsf14* ^KD^‐ID8 or *Ctrl*‐ID8 cells (Figure 7B). In summary, TNFRSF14/HVEM expression in tumor cells promotes Stat5 and Stat6 activation.

## 4 Discussion

**Figure 8:**
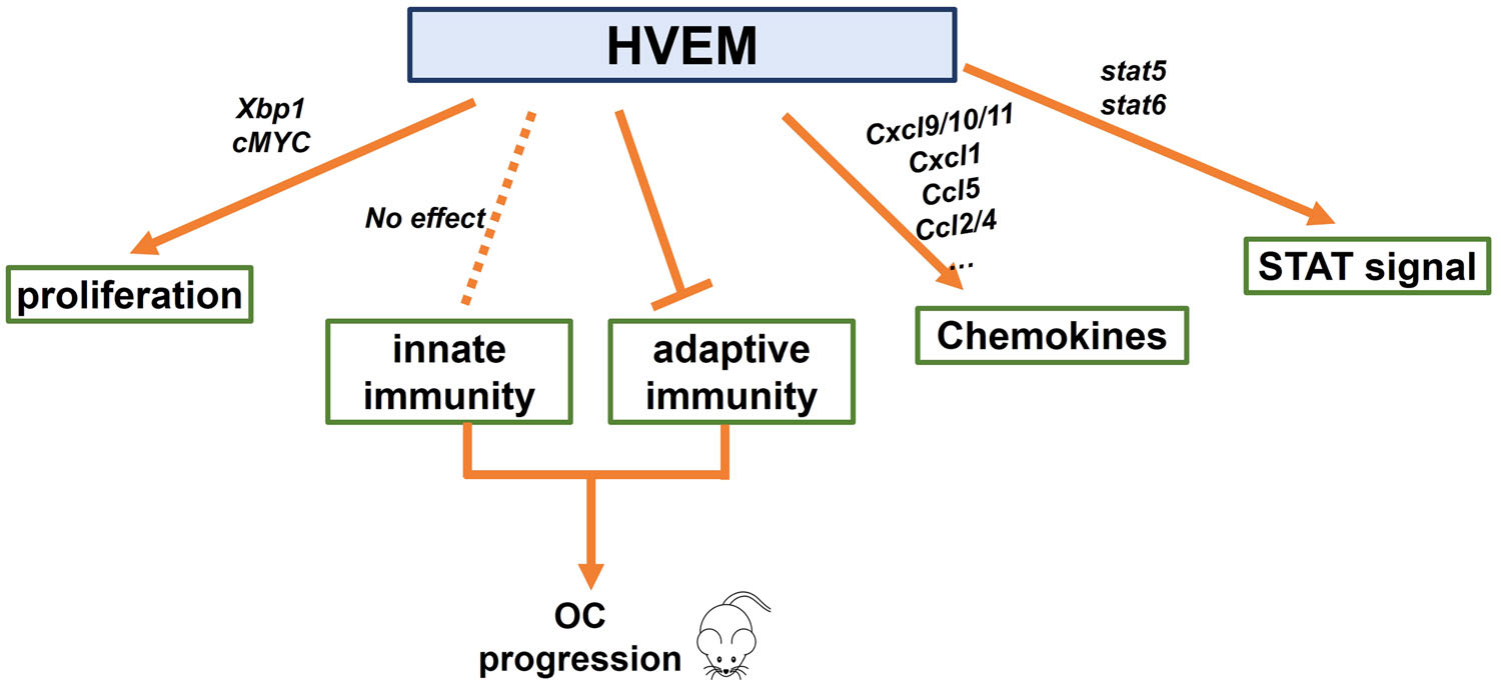
Model depicting the regulatory role of TNFRSF14/HVEM in OvCa. (1) Tumor cell‐expressed HVEM was found to promote OvCa cell proliferation by partly activating XBP1s‐cMyc signaling. (2) Tumor cell‐expressed HVEM specifically affected the effects of adaptive immunity and contributed to OvCa progression in mice. 3) Tumor cell‐expressed HVEM altered chemokine expression and STAT signal activation in ID8 cells. HVEM, herpesvirus entry mediator; OvCa, ovarian cancer; TNFRSF14, tumor necrosis factor receptor superfamily member 14.

Although immunotherapy for various types of cancer has rapidly and revolutionized development, response rates among patients with OvCa who receive immunotherapy, such as immune checkpoint inhibitor treatment, remain modest [41]. HVEM, a newly discovered checkpoint, has emerged as a potential therapeutic target for cancer treatment because of its ability to regulate lymphocyte activation and/or homeostasis [42]. In the present study, we aimed to identify the role of tumor cell‐expressed HVEM in promoting OvCa. Ectopically elevated HVEM not only promoted cancer cell proliferation but also altered the function of T cells, eventually accelerating OvCa progression (Figure 8).

HVEM has an extensive expression profile. Under physiological conditions, HVEM is observed in numerous organs, such as the endocrine tissues, gastrointestinal tract, liver, gallbladder, kidney, and urinary bladder, but is almost undetectable in the ovary at the protein level (https://www.proteinatlas.org/). HVEM is also strongly expressed in immune, mesenchymal, and epithelial cells [43, 44, 45]. Previous studies and our data have verified the upregulated mRNA levels of *TNFRSF14* in OvCa tissues and primary OvCa cells compared to those in benign tissues [23]. It should be noted that the number of cases between the OvCa and control groups in our current study exhibited a notable disparity, and further studies with a similar sample size between these two groups may make our findings more convincing. In a co‐immunofluorescence study of HVEM expression in melanoma metastases from five patients, researchers revealed that HVEM expression was mainly observed in melanoma cells, and HVEM⁺ melanoma cells were found to be contiguous with BTLA⁺ CD8⁺ T cells [46]. Similarly, in our study, we analyzed four OvCa specimens and found that HVEM expression in CD45⁻EpCAM⁺ cancer cells was higher than that in CD45⁺EpCAM⁻ immune cells in three OvCa tissues, indicating that epithelial cells are the predominant cell components with elevated expression of HVEM in OvCa specimens. The histological subtypes of the three OvCa specimens were high‐grade serous carcinoma, low‐grade serous carcinoma, and adenocarcinoma, all of which are epithelial cancers. Hence, we speculated that the dominant expression of HVEM in CD45^−^EpCAM⁺ cancer cells but not in CD45⁺EpCAM^−^ immune cells in the tumor of OvCa widely exists in different types of epithelial OvCas. However, more specimens must be collected to reach a more convincing conclusion.

To our knowledge to date, the role of HVEM expressed in cancer cells in cancer biology remains largely unknown. It is widely recognized that one of the fundamental hallmarks of cancer cells is their ability to sustain continuous proliferation [30]. Silencing *TNFRSF14* in esophageal cancer cells inhibits proliferation in vitro [47], indicating a direct role for tumor‐expressing HVEM in promoting proliferation. However, silencing HVEM in the human OvCa cell line OVCAR3 did not affect proliferation in vitro under normoxic conditions [23] but inhibited cell proliferation under hypoxic conditions [24]. Thus, the role of HVEM in promoting cancer cell proliferation remains uncertain. Our data demonstrated that the knockdown of *Tnfrsf14* in the murine OvCa cell line ID8 inhibited cancer cell proliferation in vitro under normoxic conditions. Subsequently, we revealed for the first time a novel mechanism by which HVEM enhances XBP1 activation and c‐Myc expression to promote ID8 cell proliferation. XBP1 depletion in tumor cells has been shown to inhibit cell proliferation [48]. However, the role of XBP1 in OvCa cells in cancer biology remains largely unknown. Importantly, we restored the level of spliced XBP1 (XBP1s) in *Tnfrsf14* ^KD^‐ID8 cells by treating them with a highly selective, nontoxic IRE1/XBP1s activator, IXA4, and found a partially rescued expression of c‐Myc, as well as a modestly elevated population of proliferative cells. c‐Myc is an oncogenic transcription factor that affects diverse cellular processes, including cell proliferation [49]. The regulation between XBP1s and c‐Myc may be bidirectional. Although c‐Myc has been identified as a direct downstream target of XBP1 for regulating NK cell proliferation [50], studies have also shown that MYC interacts with the b‐ZIP domain of XBP1s and increases its transcriptional activity [51]. It would be interesting to determine whether c‐Myc restoration in *Tnfrsf14* ^KD^‐ID8 cells affects XBP1s expression and cell proliferation.

However, little is known about the immunoregulation of HVEM expressed by tumor cells in the OvCa microenvironment. We observed a decreased proportion of immunosuppressive cells (MDSCs and Tregs) and an increased proportion of immunoreactive cells (NKs, NKTs, effector CD4⁺ T cells, and IFNγ‐producing CD4⁺ and CD8⁺ T cells) in the ascites of *Tnfrsf14* ^KD^‐ID8 tumor‐bearing mice, indicating a robust antitumor effect of targeting HVEM on murine OvCa. The chemokine network is key to understanding the tumor microenvironment. Our results demonstrated decreased *Ccl2, Ccl5*, and *Cxcl1* expression in *Tnfrsf14* ^KD^‐ID8 cells. In tumors, cancer cell‐derived CCL2 and CCL5 usually contribute to the recruitment of MDSCs, TAMs, and Tregs to the tumor niche [52, 53, 54, 55, 56]. Cancer cell‐derived CXCL1 also contributes to the recruitment of MDSCs [57]. Notably, a decreased proportion of ascitic MDSCs and Tregs, but not of ascitic TAMs, was observed in *Tnfrsf14* ^KD^‐ID8 tumor‐bearing mice, which likely reflects inhibited recruitment of ascitic MDSCs and Tregs by *Tnfrsf14* ^KD^‐ID8‐produced chemokines. CXCL9, CXCL10, and CXCL11 are selective ligands for the C‐X‐C motif chemokine receptor 3 (CXCR3) and are secreted by cancer cells. CXCR3 ligands in the tumor environment mainly function in two ways: recruitment of antitumor cells (CTLs, NKs, NKTs, and Th1) and contribution to the proliferation and metastasis of cancer cells [58]. Unfortunately, our results showed decreased *Cxcl9/10/11* expression in *Tnfrsf14* ^KD^‐ID8 cells and an elevated proportion of ascitic CTLs, NKs, NKTs, and Th1 cells in *Tnfrsf14* ^KD^‐ID8 tumor‐bearing mice. Hence, the downregulated expression of *Cxcl9/10/11* is more likely related to the inhibition of cancer cell proliferation and other soluble factors in the microenvironment may be involved in the accumulation of antitumor cells in ascites.

To explore the signaling pathways affected by HVEM is highly significant. HVEM can provide pro‐survival and proliferative signals through activation of NF‐κB and AKT transcriptional pathways [59]. HVEM knockdown downregulates the expression of phosphorylated AKT and mTOR in OVCAR3 cells and primary OvCa cells [24]. Unfortunately, we failed to observe obvious changes in phospho‐NF‐κB p65‐Ser536 expression in ID8 cells after silencing HVEM expression. Accumulating evidence indicates that aberrant activation of STAT5 and STAT6 signaling promotes the expression of target genes, increasing cell proliferation, survival, and metastasis in various cancers [60, 61, 62]. Herein, we observed the downregulation of phospho‐Stat5 (Tyr694) and phospho‐Stat6 (Tyr641) in *Tnfrsf14* ^KD^‐ID8 cells compared with that in *Ctrl*‐ID8 cells. Our findings suggest that HVEM influences OvCa progression by activating STAT5 and STAT6 signaling. However, the effects of the HVEM‐STAT axis in OvCa remain unclear. A lot of research must be conducted in the future.

The current study had some limitations. A notable disparity was observed in the number of cases between the OvCa and control groups. Further research with comparable sample sizes in both groups may enhance the robustness of our findings. Additionally, the current study was limited by an insufficient sample size based on the specimens (Figure 1A: power = 0.6; Figure 2H: power = 0.5). The findings from both mouse models and cellular experiments should be validated using a larger cohort of human OvCa samples to confirm their clinical relevance. Last but not least, epithelial OvCa has diverse histological subtypes. Investigations across different subtypes will enhance the potential therapeutic targeting value of HVEM.

In conclusion, our study shows that dysregulated HVEM in cancer cells promotes proliferation and provides new insights into the role of HVEM in cancer immune modulation in OvCa. Thus, HVEM may serve as a promising target for treating patients with OvCa through the regulation of cell proliferation and adaptive immunity. For future studies, it is necessary to increase the sample size by including larger and more diverse patient cohorts to obtain a more comprehensive understanding of the potential benefits and limitations of targeting HVEM in patients with OvCa.

## Author Contributions

**Yun Lu:** conceptualization, data curation, formal analysis, investigation, validation, visualization, writing – original draft, writing – review and editing. **Yijun Zhang:** investigation, validation, writing – review and editing. **Wenxuan Li:** investigation, validation. **Haonan Jiang:** data curation, investigation, software, visualization. **Jiapo Wang:** investigation, validation. **Xiaoqing Guo:** conceptualization, data curation, funding acquisition, project administration, resources, supervision, writing–review and editing.

## Ethics Statement

The study was approved by the Research Ethics Committee of Shanghai First Maternal and Infant Hospital (Ethic code: KS22274) and performed according to the Helsinki Declaration.

## Consent

All the patients were provided with written informed consent.

## Conflicts of Interest

The authors declare no conflicts of interest.

## Supporting information

Supporting information.

Supporting information.

Supporting information.

## Data Availability

The data that support the findings of this study are available from the corresponding author upon reasonable request.
