# Supplementary figures and images for: Tumor Cell‐Expressed Herpesvirus Entry Mediator Regulates Proliferation and Adaptive Immunity in Ovarian Cancer
Source: Immun Inflamm Dis. 2025 Mar 19;13(3):e70175. doi: 10.1002/iid3.70175 (PMC11921469; doi:10.1002/iid3.70175)

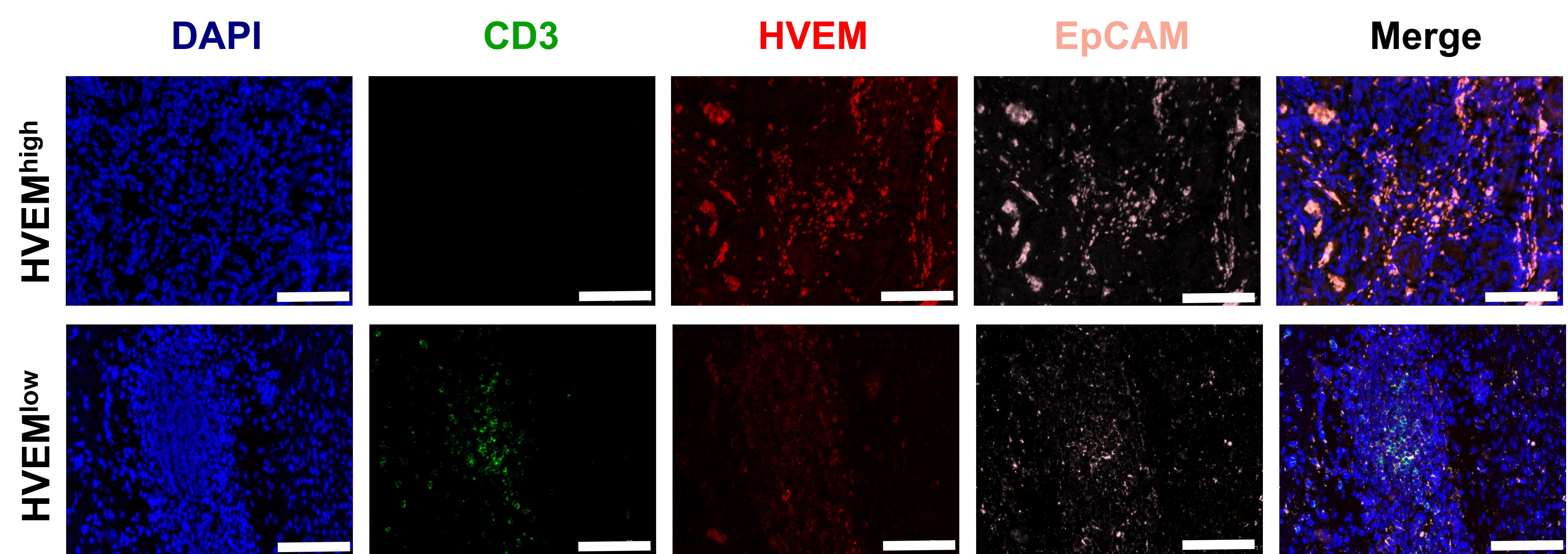

Supplement: Supplementary file 1 — Supporting information. [file IID3-13-e70175-s002.jpg]
