# Supplementary material for: Tumor Cell‐Expressed Herpesvirus Entry Mediator Regulates Proliferation and Adaptive Immunity in Ovarian Cancer
Source: Immun Inflamm Dis. 2025 Mar 19;13(3):e70175. doi: 10.1002/iid3.70175 (PMC11921469; doi:10.1002/iid3.70175)
Supplement: Supplementary file 2 — Supporting information. [file IID3-13-e70175-s001.docx]

**Table S1. Primers used in qPCR assay.**

|  | **Forward Primer (5' - 3')** | **Reverse Primer (5' - 3')** |
| --- | --- | --- |
| *ACTB* | GCGAGAAGATGACCCAGATC | CCAGTGGTACGGCCAGAGG |
| *TNFRSF14* | GTGCAGTCCAGGTTATCGTGT | CACTTGCTTAGGCCATTGAGG |
| *XBP1* | CCCTCCAGAACATCTCCCCAT | ACATGACTGGGTCCAAGTTGT |
| *Actb* | TGTCCACCTTCCAGCAGATGT | AGCTCAGTAACAGTCCGCCTAGA |
| *Tnfrsf14* | CAGGCCCCTACAGACAACAC | ACTCGTCTCCCACAAGGAACT |
| *Tnfrsf10* | ATGATGGTGATTTGCATAGTGCT | ATGATGGTGATTTGCATAGTGCT |
| *Ccl5* | GCTGCTTTGCCTACCTCTCC | TCGAGTGACAAACACGACTGC |
| *Cxcl10* | CCAAGTGCTGCCGTCATTTTC | GGCTCGCAGGGATGATTTCAA |
| *Cxcl1* | ACTGCACCCAAACCGAAGTC | TGGGGACACCTTTTAGCATCTT |
| *Il15ra* | ATGATGGTGATTTGCATAGTGCT | ATGATGGTGATTTGCATAGTGCT |
| *Il2rg* | CTCAGGCAACCAACCTCAC | GCTGGACAACAAATGTCTGGTAG |
| *Csf1* | ATGAGCAGGAGTATTGCCAAGG | TCCATTCCCAATCATGTGGCTA |
| *Cxcl9* | GGAGTTCGAGGAACCCTAGTG | GGGATTTGTAGTGGATCGTGC |
| *Rac2* | GACAGTAAGCCGGTGAACCTG | CTGACTAGCGAGAAGCAGATG |
| *Ccl2* | TTAAAAACCTGGATCGGAACCAA | GCATTAGCTTCAGATTTACGGGT |
| *Ccl4* | TTCCTGCTGTTTCTCTTACACCT | CTGTCTGCCTCTTTTGGTCAG |
| *Cxcl11* | GGCTTCCTTATGTTCAAACAGGG | GCCGTTACTCGGGTAAATTACA |
| *Was* | ACCAGCACCACAGTATTCAAATG | TTGTTTTCAGTGCAGACCGAT |
| *Hck* | CCACCAAAGGGAGCTACTCG | GCCCATCCTTCCCCTTCTTG |
