# Supplementary material for: Tumor Cell‐Expressed Herpesvirus Entry Mediator Regulates Proliferation and Adaptive Immunity in Ovarian Cancer
Source: Immun Inflamm Dis. 2025 Mar 19;13(3):e70175. doi: 10.1002/iid3.70175 (PMC11921469; doi:10.1002/iid3.70175)
Supplement: Supplementary file 3 — Supporting information. [file IID3-13-e70175-s003.docx]

**Figure S1. CD3^+^ T cell infiltration in specimens from OvCa patients.** Representative immunofluorescent staining of HVEM, CD3, EpCAM, and DAPI for specimens from OvCa patients (n = 40). Scale bar: 100μm.
